# Maternal onset *de novo* SH2D1A mutation and lymphocytic choriomeningitis virus infection in a patient with X-linked lymphoproliferative disease type 1: A case report

**DOI:** 10.3892/mmr.2015.3173

**Published:** 2015-01-09

**Authors:** JINRONG LIU, WENJUN TIAN, FANG WANG, WEN TENG, YANG ZHANG, CHUNRONG TONG, CHONGLIN ZHANG, YING JU, BINGCHANG ZHANG, SHUNYING ZHAO, HONGXING LIU

**Affiliations:** 1 Department of Respiratory and Infectious Diseases, Beijing Children’s Hospital of Capital Medical University, Beijing 100045, P.R. China; 2 Department of Clinical Laboratory, Shandong Provincial Hospital Affiliated to Shandong University, Jinan, Shandong 250021, P.R. China; 3 Department of Molecular Genetics Laboratory, Beijing Daopei Hospital, Beijing 100049, P.R. China; 4 Department of Respiratory, Xuzhou Children’s Hospital, Xuzhou, Jinagsu 221002, P.R. China

**Keywords:** X-linked lymphoproliferative disease type 1, SH2D1A mutation, lymphocytic choriomeningitis virus, *de novo* mutation

## Abstract

X-linked lymphoproliferative disease type 1 (XLP1) is a rare genetic immunodeficiency disease, which occurs due to germline mutations in the SH2D1A gene. This gene has been reported to encode the adaptor molecule signaling lymphocytic activation molecule-associated protein XLP1 is generally triggered by the Epstein-Barr virus (EBV) infection. The present study reported the case of a 4-year-old male who presented with a high fever, hypogammaglobulinemia, diffuse lung disease and encephalitis. The patient was infected with the lymphocytic choriomeningitis virus (LCMV), not EBV or any other human herpes virus. The patient was found to carry a SH2D1A c.7G>T/p.A3S mutation, which was inherited from the mother and maternal grandfather, as well as a SH2D1A c.228T>A/p.Y76X mutation, which was identified to be a maternal-onset *de novo* mutation at the time of germline development of the patient’s mother. To the best of our knowledge, the present study is the first reported case of maternal-onset XLP1 with a *de novo* SH2D1A mutation and LCMV infection.

## Introduction

X-linked lymphoproliferative disease type 1 (XLP1) is a well-defined maternally-inherited immunodeficiency disease caused by a mutation of the SH2D1A gene, which encodes for signaling lymphocytic activation molecule (SLAM)-associated protein (SAP), leading to functional defects in natural killer (NK) cells, CD8^+^ T cells and natural killer T (NKT) cells (1). Clinical manifestations of this genetic mutation vary, the most severe complications may include hemophagocytic lymphohistiocytosis (HLH), lymphoproliferative disorders, dysgammaglobulinemia and vasculitis, which are often triggered by Epstein-Barr virus (EBV) infection (2). The present study reported the case of a 4-year-old male patient with a maternal-onset *de novo* SH2D1A mutation, who had suffered from lymphocytic choriomeningitis (LCMV) infection and developed XLP1, together with other atypical clinical manifestations.

## Case report

**Figure 1:**
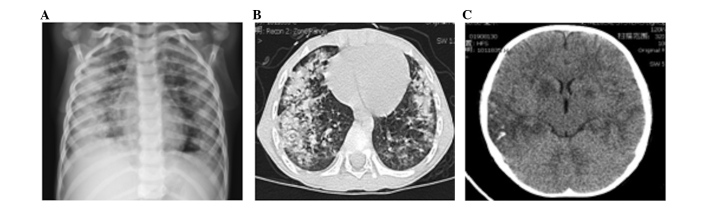
Chest X-ray and CT scan of the patient. (A) Chest X-ray revealing diffuse lung disease. (B) Lung CT scan showing diffuse parenchymal infiltration. (C) Head CT scan showing multiple low density areas in the left frontal lobe, right temporal lobe, parietal lobe, left parietal-occipital lobe, bilateral basal ganglia and trigone of the left lateral ventricle, with bleeding in certain areas. CT, computerized tomography.

The patient was a 4 year-old male who had experienced intermittent high fevers and a mild cough for six weeks. On physical examination the patient present with tachypnea, but no other respiratory symptoms and the examination of the central nervous system was normal. The study was approved by the ethics committee of Shandong Provincial Hospital Affiliated to Shandong University (Jinan, China). Written informed consent was obtained from the patient’s family. Laboratory testing revealed hypogammaglobulinemia, elevated C-reactive protein levels and mild pancytopenia. Sputum and blood cultures were negative for bacteria, fungi and *Mycobacterium tuberculosis*. Examinations, including those for the serum fungal G test, galactomannan test, toxoplasma antibodies, cryptococcal antigens, anti-nuclear antibodies, double-stranded DNA, anti-neutrophil cytoplasmic antibodies and extractable nuclear antigens, were all negative. In addition, morphological examination of the bone marrow was normal. Chest X-ray and computerized tomography (CT) scans revealed diffuse lung infiltrates (Fig. 1A and B). Following admission, the patient was treated with ganciclovir and a γ-globulin (400 mg/kg) infusion. High fevers and coughing continued, and the patient developed lethargy, slurred speech and convulsions. A head CT scan revealed multiple low density areas in the left frontal lobe, right temporal lobe, parietal lobe, left parietal-occipital lobe, bilateral basal ganglia and trigone of the left lateral ventricle, with bleeding in the right temporal lobe (Fig. 1C). The patient succumbed to a pulmonary hemorrhage two weeks following admission. The patient had been exposed to a cat in the home environment since birth and had a history of recurrent respiratory tract infections.

### Detection of the human herpes virus (HHV) and LCMV

Plasma DNA was screened for HHV 1-8 virus infections using multiplex polymerase chain reaction (PCR), as previously described (3,4), the results of which were negative. In addition, reverse transcription-quantitative PCR was performed, as previously described (5), in order to quantitatively analyze LCMV in plasma. The results revealed that the LCMV load was 7.8×10^5^ copies/ml at the time of admission and 2.3×10^6^ copies/ml on the eleventh day of admission when the patient began to develop lethargy, slurred speech and convulsions.

### SH2D1A gene mutation, pedigree analysis and clone sequencing analysis

Genomic DNA extracted from peripheral blood mononuclear cells (PBMCs) on the second day of admission was used for analysis and primers were designed to amplify the coding sequence of the SH2D1A gene. The PCR product was sequenced by Sanger sequencing using an AB3130XL genetic analyzer (Applied Biosystems^®^, Life Technologies, Grand Island, NY, USA) and mutations were analyzed using VariantReporter V1.0 software (Life Technologies).

**Figure 2:**
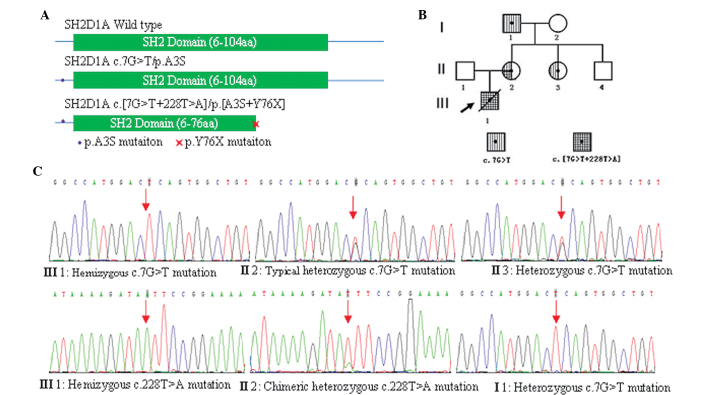
SH2D1A gene mutation, pedigree analysis and clone sequencing analysis. (A) SH2D1A gene encodes the 128-amino acid protein SAP. Polymerase chain reaction amplification and sequencing of the patient’s DNA revealed that the p.A3S mutation was located at the N-terminal sequence of SAP and p.Y76X mutation was predicted to truncate SAP at tyrosine 76; therefore, greatly interfering with normal SAP function. (B) Pedigree of three generations of the patient’s family for SH2D1A gene mutation(s). Circles represent females, while squares indicate males. Arrow signifies the proband and slash through symbol indicates mortality. (C) Sequencing results of amplified genomic DNA from peripheral blood mononuclear cells from the proband and their family showing: hemizygous c.7G>T and c.228T>A mutations in the proband (III1); typical heterozygous c.7G>T mutation and chimeric heterozygous c.228T>A mutation in II2, heterozygous c.7G>T mutation in II 3 and hemizygous c.7G>T mutation in I1. SAP, signaling lymphocytic activation molecule-associated protein; III1, proband; II1 and II2, proband’s father and mother, respectively; II3 and II4, mother’s sister and brother, respectively; I1 and I2, proband’s maternal grandfather and grandmother, respectively.

PCR amplification and sequencing results demonstrated that the proband carried both hemizygous SH2D1A c.7G>T/p.A3S and c.228T>A/p.Y76X mutations and the p.Y76X mutation was predicted to truncate SAP at tyrosine 76 (p.Y76X) (Fig. 2A). The two mutations were found to be carried by the proband’s mother, with the c.7G>T mutation showing a typical heterozygous profile (Fig. 2B and C); however, the c.228T>A mutation showed a chimeric heterozygous profile (Fig. 2C). The sister of the proband’s mother carried only a heterozygous c.7G>T mutation (Fig. 2B and C) and their brother was not a carrier for either mutation (Fig. 2B). The proband’s maternal grandfather was also found to carry the hemizygous c.7G>T mutation, but not the c.228T>A mutation (Fig. 2B and C). Neither mutation was carried by the proband’s father or maternal grandmother.

Primers were designed for amplification of the SH2D1A mRNA sequence, including the c.7G>T and c.228T>A mutation loci. In addition, reverse transcription-PCR was performed using total RNA extracted from PBMCs. The PCR product was purified, cloned and then sequenced by Sanger sequencing. The results showed that the two mutations were located on the same SH2D1A allele in the proband’s mother, with 26/96 clones of the SH2D1A mRNA molecules carrying the two mutations and 23/96 clones only carrying the c.7G>T mutation. Clone sequencing of the genomic DNA PCR product also revealed that 17/36 clones carried the c.7G>T mutation and 13/49 clones carried the c.228T>A mutation. Genotyping of all 15 short tandem repeat (STR) loci, using an AB Identifier forensic kit, demonstrated that the proband’s mother exhibited one or two genotypes at each STR loci, indicating that she was not a chimera.

## Discussion

XLP1 is a rare and often fatal X-linked immunodeficiency disease caused by mutation of the SH2D1A gene, this gene encodes for a 128-amino acid protein (SAP), which is comprised of a 5-amino acid N-terminal sequence, a Src homology 2 (SH2) domain and a 25-amino acid C-terminal tail (6). To date, all reported families with XLP1 have followed the regular inheritance pattern of X-linked genetic disease. Furthermore, the reported probands carried only one detrimental SH2D1A mutation or partial/whole SH2D1A gene deletion, which was often triggered by an EBV infection (1,7).

In the present case report, the proband carried two mutations in a single maternally inherited SH2D1A allele. The p.A3S mutation was found to be inherited from the maternal grandfather, not from the maternal grandmother, and was located at the N-terminal of the SAP protein, outside of the SH2 domain (Fig. 2A). The proband’s maternal grandfather carried a hemizygous p.A3S mutation, but maintained a normal immune status, indicating that the p.A3S mutation was not lethal. Therefore, it was hypothesized that the p.A3S mutation may not have a significant adverse impact on the SH2 domain.

The p.Y76X mutation was predicted to truncate SAP at tyrosine 76 (p.Y76X) (Fig. 2A), thus resulting in severe deleterious effects on the function. A previous patient was reported to carry an inherited germiline p.Y76X mutation and suffered from hemophagocytic lymphohistiocytosis, hypogammaglobulinemia and EBV infection (8), which also corroborated the pathogenicity of this mutation. In the present study, direct sequencing and clone sequencing were used to determine the chimeric mutation status of the proband’s mother. The results revealed that the c.228T>A mutation was a *de novo* mutation, which occurred in the same SH2D1A c.7G>T mutated allele at the time of the first division of the fertilized egg. A previous study indicated that humans have an intergenerational mutation rate of ~1.1×10^−8^ per position per haploid genome (9). In addition, comparative genomic methods were used to estimate that any single conceptus has 1–3 novel deleterious mutations, which lead to an altered amino acid; therefore, it was predicted that on average, one novel mutation occurs every 10,000 genes/zygote (10). *De novo* mutations of rare diseases have been reported occasionally (11); however, such mutations have not been reported for XLP1. Spontaneous mutations may have severe phenotypic consequences when they functionally affect relevant bases. In addition, when these mutations are carried in germ cells, they may be inherited.

The patient in the current report had a LCMV infection, but no EBV or other HHV infection. LCMV, the prototype of the *Arenaviridae* family, has been associated with the natural reservoir *Mus domesticus*. In addition, LCMV has been reported to cause meningitis and a flu-like illness. The prevalence of LCMV in urban residents was found to be 1.0–3.6% (12). LCMV has also been shown to cause pulmonary infiltrates and central nervous system disease in humans, predominantly in people with immune deficiencies, including leukemia (13) or in post-transplantation patients (14). It was reported that LCMV infection may result in the mortality of SAP-deficient mice, indicating that SAP may have an important role in LCMV immunity (15).

In conclusion, it was therefore considered that in the present case report, the LCMV infection may have resulted in the onset of XLP1 and the aberrant clinical phenotype in the patient.
